# Social vulnerability and asthma-related emergency medical services in Illinois

**DOI:** 10.3389/fpubh.2025.1521545

**Published:** 2025-02-26

**Authors:** Sarah Dee Geiger, M. Omar Khaium, Evan M. Gustafson, Marcus Shapiro, Sarah Keeley, Cassandra Johnson, Nancy Amerson, Daniel Lee, Lynn B. Gerald, Arlene Keddie

**Affiliations:** ^1^Department of Health and Kinesiology, University of Illinois Urbana-Champaign, Champaign, IL, United States; ^2^Southern Illinois University School of Medicine, Springfield, IL, United States; ^3^Department of Occupation and Environmental Health Sciences, West Virginia University, Morgantown, WV, United States; ^4^Division of Chronic Disease, Illinois Department of Public Health, Springfield, IL, United States; ^5^Division of EMS, Illinois Department of Public Health, Springfield, IL, United States; ^6^Population Health Sciences Program, University of Illinois Chicago, Chicago, IL, United States; ^7^School of Health Studies, Northern Illinois University, DeKalb, IL, United States

**Keywords:** asthma, emergency visits, social vulnerability index, COVID-19, vulnerability

## Abstract

**Introduction:**

This ecologic study explores the relationship between the Social Vulnerability Index (SVI) and probable asthma-related emergency medical service (EMS) rates before and during the COVID-19 pandemic at the county level in Illinois.

**Methods:**

The number of asthma-related EMS visits was obtained in all 102 counties for adults aged 18 years or more, and for 82 of these counties for children aged less than 18 from 2018 to 2021. They were converted into rates and examined in relation to SVI rankings. Pearson’s correlation coefficients and negative binomial regression were used to examine associations, adjusting for the level of rurality.

**Results:**

The rate of asthma-related EMS visits increased in adults from 23.1 to 28.9 per 1,000 between 2018 and 2021 but decreased in children from 5.9 to 5 per 1,000. Every 1% increase in the overall SVI was associated with a nearly two-fold increase in the rate of EMS visits in adults (pre-COVID-19: IRR = 1.94, *p* < 0.01; during-COVID: IRR = 1.90, *p* < 0.01) and an even greater increase in children (pre-COVID-19: IRR = 2.61, *p* < 0.01; during-COVID: IRR = 2.92, *p* < 0.01) Consistent associations were found for the SVI themes of socioeconomic status, housing type, and transportation across age groups and time periods.

**Discussion:**

During the pandemic, all four themes of SVI were associated with the asthma EMS rate in children. This study has shown that the same factors that lead to community vulnerability in a disaster align with a greater need for on-site asthma emergency treatment.

## Introduction

Asthma, which impacts approximately 25 million Americans, remains a significant long-term health issue in the United States (1). A nationwide 20-year-long projection on asthma suggests that the total financial burden might be around $960 billion, or about 20% of the US economy’s annual income (2, 3). The national prevalence of asthma in 2021 was 7.7%, which was 1.0% lower than in Illinois (8.7%) (1, 4). In that year alone, asthma attacks led to 986,453 Emergency Department (ED) visits, with each one costing between $600 to $1,500, and 94,560 hospital inpatient admissions (1, 5, 6).

Asthma has a complicated etiology that involves interactions between genetic, environmental, and immune components (7). Similarly, the social and family environments can contribute to the development and exacerbation of asthma symptoms (8, 9). Sudden asthma exacerbations, the lack of appropriate education on asthma management, challenges with medication management, and asthma emergency preparedness at home, or the absence of a primary care physician may lead to increased use of emergency medical services (EMS) (10). Most of the EMS recipients for asthma are children, and reliance on these services is more common among minorities (11, 12). Emergency department (ED) visits are also common among children and minorities with lower socioeconomic status (SES) (13, 14). Low-income individuals, uninsured, or underinsured patients may delay seeking care until an emergency arises due to financial constraints, resulting in unequal care (15, 16). The presence of healthcare disparities across the nation accentuates the unequal burden of asthma, as reflected in almost two times higher asthma-related mortality rates among non-Hispanic Black people than non-Hispanic White people (1). Studies have reported that during the COVID-19 pandemic, the volume of EMS calls was significantly reduced as people may have deferred seeking care to reduce COVID-19 exposure risk (17, 18). Moreover, the EMS rates for acute exacerbation of chronic diseases, including asthma decreased due to the fear of transmission of COVID-19 (19). Therefore, we sought to examine differences across time in this study.

Management of asthma symptoms during the pandemic was laden with struggles for asthma patients and their caregivers. Not only anxiety about transmission of COVID-19 but also challenges in getting asthma medication and suitable care were unceasingly difficult early in the pandemic (20, 21). Patients opted for virtual consultations and expressed the need for more comprehensive care which was difficult to render due to the dearth of required diagnostic tests and medical procedures, healthcare staff shortage, and canceled in-hospital appointments, to name a few (22, 23). Moreover, socioeconomically disadvantaged asthma patients reported reduced use of asthma medication and proper healthcare for the disease (24). EMS personnel reported an overwhelming workload and resource constraints due to the pandemic, and finally, changes in asthma management such as a significant reduction in bronchodilator administration occurred as well (25, 26). For all of these reasons, associations between asthma-related EMS rates and social vulnerability were examined both before and during the pandemic.

**Figure 1:**
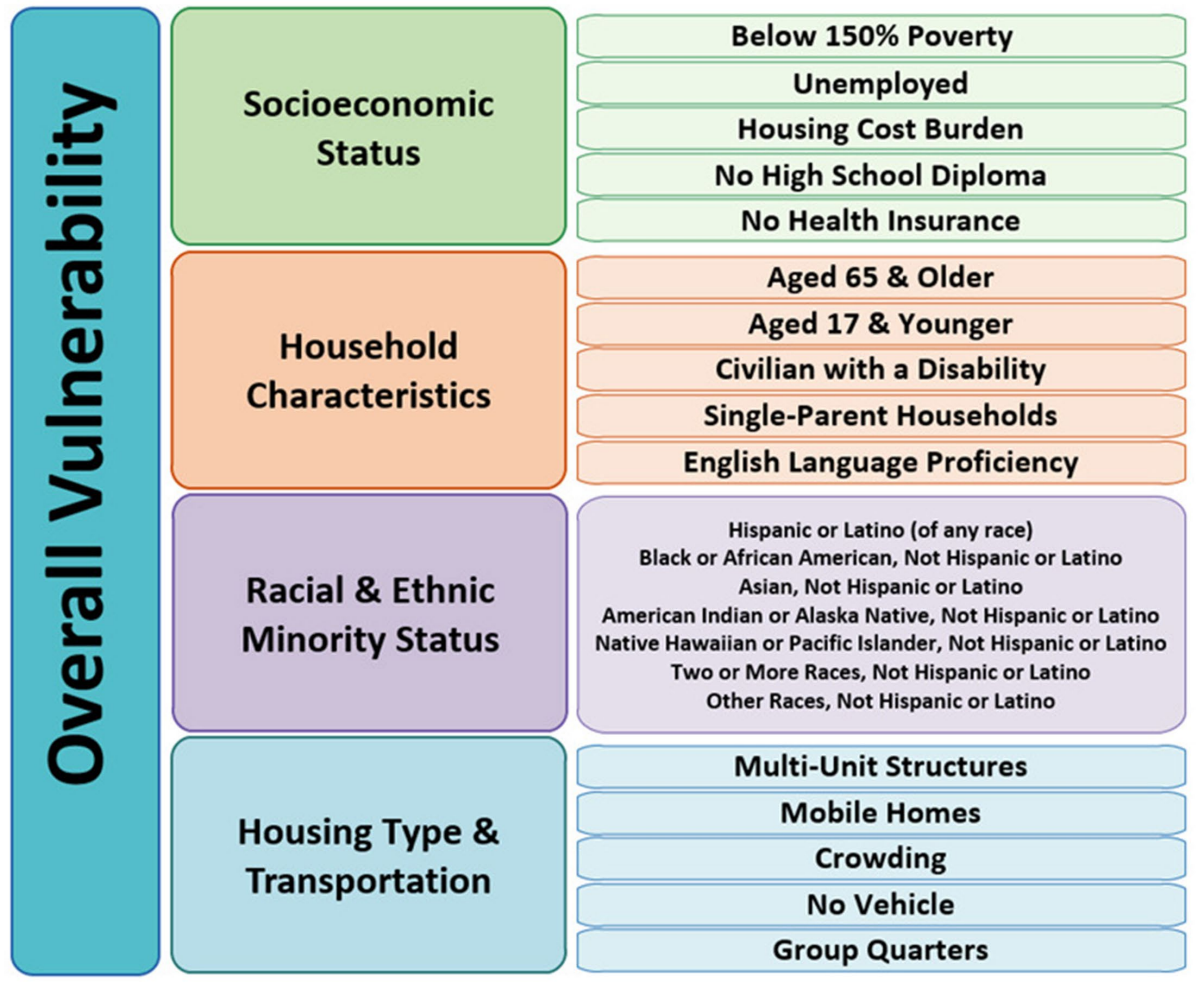
Breakdown of key themes into social factors of overall vulnerability.

One of the key concepts to determine a community’s capacity to mitigate health impacts resulting from external stressors is social vulnerability (27). The Centers for Disease Control and Prevention (CDC) and the Agency for Toxic Substances and Disease Registry (ATSDR) created the SVI to identify communities at greater risk of both financial and human suffering in the event of a disaster and to strategize assistance for communities anticipated to require support prior to, during, and after a public health emergency. However, this index may also prove useful in the identification of health issues. The SVI consists of 16 social factors grouped into four key themes: (1) Socioeconomic Status, (2) Household Characteristics, (3) Racial and Ethnic Minority Status, and (4) Housing Type and Transportation (27). All 16 social factors within these four themes are displayed in Figure 1.

Correlations between SVI and other chronic illnesses have been found in recent years (28–33). Higher social vulnerability is associated with a higher rate of ED visits overall (34). In relation to asthma, there are very few studies in which the SVI has been used. A county-level ecologic study by Nayak et al. examined and found associations between SVI and asthma-related ED visits and hospitalizations for several states excluding Illinois (35). During the COVID-19 pandemic, the number of ED visits for asthma dramatically declined (36–38). This does not necessarily indicate improved asthma control. It is possible that school closures, remote work, mandatory stay-at-home orders, and COVID-19 mitigation strategies provided protection from respiratory infections and asthma triggers (39).

Associations between asthma-related EMS rates and the SVI have never been investigated, a gap this ecologic study primarily aims to address by examining the relationship between them on the county level, both in general and more specifically based on the different themes of SVI. The secondary objectives of this study is to discern patterns of association between adults (≥ 18 years old) and children (< 18 years old) before and during the COVID-19 pandemic. Changes in EMS utilization across this period can reflect shifts in access and public health practices, mapping out the strengths and flaws of healthcare systems and providing a unique opportunity to evaluate how external stressors influence asthma management and healthcare-seeking behaviors. This study has the potential to help shape health policies by demonstrating the importance of identifying high-risk areas that may benefit from targeted asthma interventions.

## Methods

### SVI

In this ecologic study, we obtained Illinois-specific SVI county data for the years 2018 and 2020, and categorized the data into pre-COVID-19 and during-COVID-19 periods; 2018 data represents the pre-COVID-19 period, and 2020 SVI data corresponds to the COVID-19 period.

The Geospatial Research, Analysis, and Services Program (GRASP) in collaboration with the CDC and the ATSDR created the SVI in 2011 to locate communities that may be especially vulnerable during natural and manmade disasters (27). Originally designed to assist the public health community in identifying and assisting vulnerable communities during disasters or emergencies, the SVI has been discovered to correlate with various health-related indicators (40, 41).

The SVI is a robust metric to assess relative vulnerability at the county level by US census tract data. By examining 16 social factors, grouped into four themes (as shown in Figure 1), the index facilitates community assessment values and vulnerability rankings (27, 28, 41). These rankings are established based on percentiles, ranging from 0 to 1, where higher values indicate heightened vulnerability. According to the levels of vulnerability presented by CDC/ATSDR, the overall SVI metric is distributed into four levels- Low (0 to 0.25), Low-Medium (0.26 to 0.50), Medium-High (0.51 to 0.75) and High (0.76 to 1) (40).

### EMS for probable asthma

We requested age and probable asthma-specific EMS response counts from the Illinois Department of Public Health (IDPH) and calculated rates using publicly available census data covering the 4 years from 2018 to 2021. EMS rates for each county were computed by taking the total count of EMS visits for probable asthma divided by the total number at risk in the county, multiplied by 1,000. Therefore the rates are expressed per 1,000 individuals per year.

During this time period, the Illinois EMS dataset was undergoing a transition from version 2.2.1 to version 3.4 of the national standard for this type of data (NEMSIS). This transition began in the third quarter of 2016 and was completed within the next 2 years. As a result, all analyses for the years 2019 through 2021 were completed using version 3.4 data only, while 2018 analyses used a combination of version 2.2.1 and version 3.4 data.

The definition of probable asthma that we used changed slightly between the two versions. Both versions include at least one administration of inhaled albuterol and respiratory distress as part of initial assessment. In addition, version 2.2.1 is also based on breathing problems as the primary symptom. Version 3.4 includes shortness of breath, dyspnea, wheezing, and asthma with exacerbation as primary symptoms, acute respiratory disorder, acute bronchospasm, dyspnea, and ICD 10 code J45 series as primary assessment.

We stratified by pre-COVID-19 and during-COVID-19 time periods due to the influence of the pandemic on both healthcare-seeking and asthma (37, 38, 42). The years were combined primarily to constitute the pre-COVID-19 period (2018 and 2019), and the during-COVID-19 period (2020 and 2021). Single-year county-level EMS rates were statistically unstable due to low numbers. SVI data was available for the years 2018 and 2020, which nearly matches the time period of the EMS rates data.

The number of EMS visits per county was sufficient to use all 102 counties in the adult analyses. We defined adults as people aged 18 or over, and children as those under 18. Twenty counties had fewer than five EMS visits to children for the 2 years combined which were excluded, meaning that only 82 counties were used for the child analyses. We conducted sensitivity analyses by excluding counties with less than 10, 15, and 20 asthma-related EMS visits per year, and the results were consistent across models.

### Rurality

The geographical distribution within Illinois primarily comprises rural counties, accounting for 83.3% of the state’s land area, with urban counties constituting the remaining portion. The rurality criterion relied on IDPH classifications, where rural is described as a county that is either not included in a metropolitan statistical area (MSA) according to the U.S. Census Bureau’s definition or is part of an MSA but has a population of less than 60,000 (43). Considering that distance from a hospital could influence the frequency of EMS visits, we adjusted for rurality in our regression analysis.

### Statistical analysis

Data analysis was conducted using the statistical software SAS (OnDemand for Academics) version 9.4 M7 (44). Prior to analysis, all variables were examined for distribution and dispersion characteristics. To assess the relationship between the SVI and EMS rates, Pearson’s Correlation Coefficients were calculated, with a significance level set at *p* < 0.05.

Considering that the EMS data represents count data exhibiting overdispersion (Table 1), negative binomial regression was chosen as the most appropriate model to explore potential associations between the overall SVI and its themes, and EMS counts while accounting for the influence of rurality and county population (as the offset variable). A diagnostic test for overdispersion, Dispersion Statistic, was carried out in PROC GENMOD. All the components of SVI were statistically significant in the test, as shown in Table 1, suggesting overdispersion exists in the data.

**Table 1:** Significant test for overdispersion (**ꭓ**^2^).

Model	Overall SVI^a^	Socioeconomic status	Household characteristics	Racial and ethnic minority status	Housing type and transportation
Children, pre-COVID-19	130.43***	140.63***	150.30***	96.19***	160.17***
Children, during COVID-19	236.91***	242.75***	258.8***	119.7***	170.92***
Adults, pre-COVID-19	1035.76***	1053***	1178.64***	610.71***	1052.31***
Adults, during COVID-19	1915.04***	2063.58***	1891.02***	991.38***	1832.64***

a SVI: Social Vulnerability Index.

*Statistically significant (*p*-value < 0.05). **Statistically significant (*p*-value < 0.01). ***Statistically significant (*p*-value < 0.001).

## Results

Concerning probable asthma-related adult Emergency Medical Service rates, the pre-COVID-19 rate was 23.1 (95% CI [21.11, 25.10]) per 1,000 individuals, which subsequently increased to 28.9 (95% CI [26.58, 31.23]) per 1,000 during the COVID-19 period. Among Illinois children, the pre-COVID-19 EMS rate was 5.9 (95% CI [5.14, 6.58]) per 1,000, which decreased to 5.0 (95% CI [4.39, 5.63]) per 1,000 during the pandemic.

**Figure 2:**
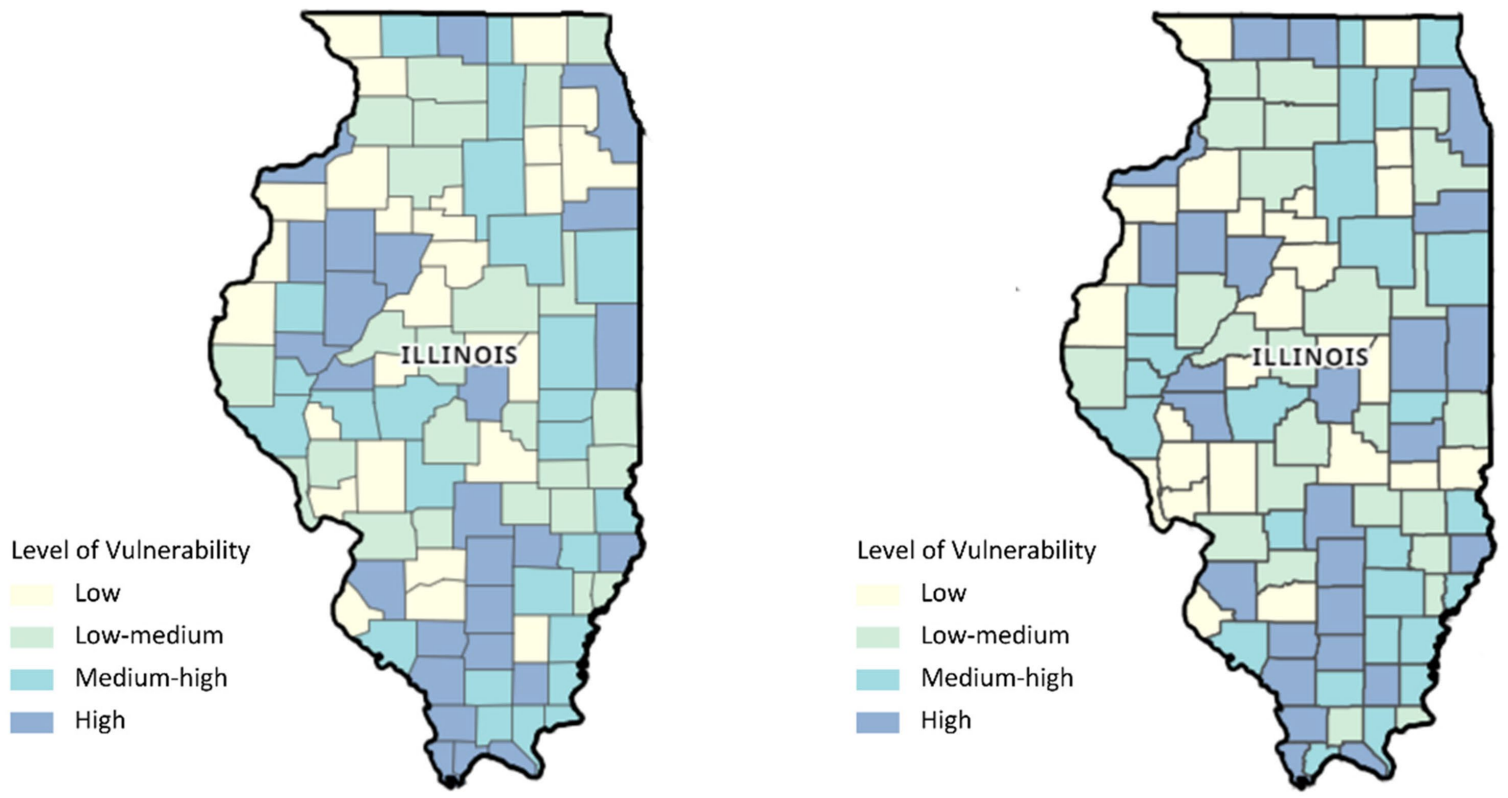
Map of Illinois showing the distribution of the Social Vulnerability Index (SVI) for 2018 and 2020 (from the left).

**Figure 3:**
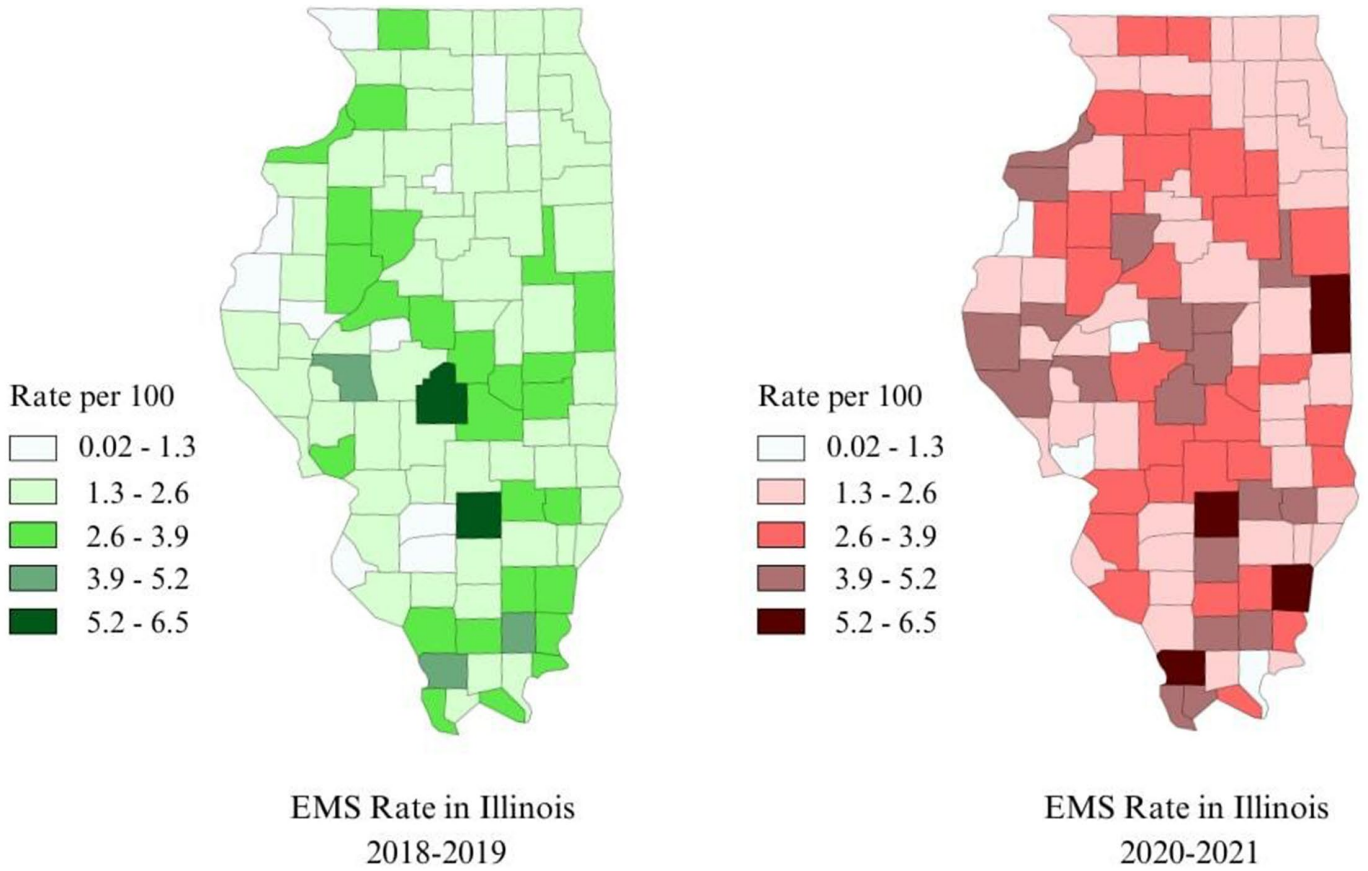
Map of Illinois showing the distribution of Emergency Medical Services (EMS) for asthma in Illinois for 2018 and 2020 (from the left).

Figure 2 shows the side-by-side distribution of overall SVI among the counties of Illinois according to the levels of vulnerability for pre-COVID-19 and during COVID-19 whereas Figure 3 displays the EMS rate for asthma during each of the two time periods. Viewing the two figures in combination, counties with high EMS rates also tended to have high SVI ranks, and vice versa. For instance, 75% of the 5 counties with high EMS rates (≥ 4 per 100) before the COVID-19 pandemic had an SVI in the range of medium-high to high level of vulnerability (0.5–1) in 2018. Out of 29 counties with moderate to high EMS rates (2.7–6.4 per 100), 20 (69%) were in the range of medium-high to high level (0.5–1) of social vulnerability. Turning to the 20 counties with the lowest level of EMS rates (0–1.5 per 100), 12 of them (60%) also had SVI scores among the least vulnerable (0–0.25) (30).

During the COVID-19 pandemic, 41 counties experienced moderate to high EMS rates (3.1–6.02 per 100), out of which 28 (68%) were in the range of medium-high to high levels of vulnerability (0.5–1), in 2020. Likewise, 7 out of 9 counties (78%) with the lowest EMS rates (0–1.5 per 100) ranked among the least vulnerable (0–0.25).

### Correlation between county-level SVI and EMS rates for adults and children

Significant correlations between SVI and probable asthma EMS rates were observed among adults throughout Illinois. For overall SVI, a statistically significant positive correlation remained essentially unchanged in both the pre-COVID-19 period (*r* = 0.42, *p* < 0.01) and during the pandemic period (*r* = 0.42, *p* < 0.01), as shown in Table 2. Three SVI themes demonstrated moderately positive and statistically significant correlations both before and during COVID-19: SES, household characteristics, and housing type and transportation. However, there was no significant correlation between the minority status theme and probable asthma EMS rates among adults in either period.

**Table 2:** Correlation analysis for SVI and asthma-related EMS rates by age and time period.

SVI^a^	Adult (County *n* = 102)		Youth (County *n* = 82)	
	Pre-COVID-19	During-COVID-19	Pre-COVID-19	During-COVID-19
	Pearson’s *r*	Pearson’s *r*	Pearson’s *r*	Pearson’s *r*
Overall	0.42***	0.42***	0.46***	0.52***
SES^b^	0.39***	0.36***	0.42***	0.42***
Household characteristics	0.39***	0.37***	0.09	0.28**
Minority status	−0.08	0.11	0.19	0.44***
Housing type and transportation	0.35***	0.36***	0.48***	0.48***

a SVI: Social Vulnerability Index.

b SES: Socioeconomic status.

*Statistically significant (*p*-value < 0.05). **Statistically significant (*p*-value < 0.01). ***Statistically significant (*p*-value < 0.001).

Similar findings were observed for children in Illinois. Overall, SVI for both the pre-COVID-19 period (*r* = 0.46, *p* < 0.01) and the COVID-19 period (*r* = 0.52, *p* < 0.01) showed a moderately positive and statistically significant relationship (Refer to Table 2.). In the pre-COVID-19 period, SES, and housing type/transportation were significantly correlated with probable asthma EMS rates, while during the pandemic, all themes showed significant positive correlations.

### Negative binomial regression analysis

Negative binomial regression analysis for the adult population prior to the COVID-19 pandemic revealed significant associations between SVI and probable asthma EMS rates. In the pre-COVID-19 period, a one-unit (1 %) increase in overall SVI was associated with a 94% increase in the probable asthma EMS rate (IRR 1.94, 95% CI [1.49, 2.53], *p* < 0.05). Similarly, during the COVID-19 period, with each percentage increase in overall SVI the EMS rate increased by 90% (IRR 1.90, 95% CI [1.43, 2.53], *p* < 0.05) compared to areas with a lower SVI, assuming all other factors remain constant. During the COVID-19 period, the relationship between socioeconomic status, household characteristics, housing type/transportation, and EMS rate remained relatively similar to the pre-COVID-19 period. However, there was a noteworthy change in association with minority status, although it still did not reach statistical significance (see Table 3).

**Table 3:** Negative binomial regression analysis for SVI and asthma-related EMS rates of adults.

SVI^a^	Pre-COVID-19			During-COVID-19		
	IRR^b^	SE^c^	95% CI^d^ (Lower, Upper)	IRR	SE	95% CI (Lower, Upper)
Overall	1.94***	0.14	(1.49, 2.53)	1.90***	0.15	(1.43, 2.53)
SES^e^	1.86***	0.14	(1.40, 2.45)	1.70	0.15	(1.27, 2.27)
Household Characteristics	1.72***	0.14	(1.32, 2.26)	1.66	0.14	(1.26, 2.19)
Minority Status	0.95	0.18	(0.67, 1.34)	1.35	0.18	(0.94, 1.94)
Housing Type and Transportation	1.85***	0.14	(1.40, 2.45)	1.79***	0.15	(1.33, 2.40)

a SVI: Social Vulnerability Index.

b IRR: Incident Rate Ratio.

c SE: Standard Error.

d CI: Confidence Interval.

e SES: Socioeconomic Status.

*Statistically significant (*p*-value <0.05). **Statistically significant (*p*-value <0.01). ***Statistically significant (*p*-value <0.001).

Similarly, among children, overall SVI demonstrated significant predictive power for probable asthma-related EMS visits. In the pre-COVID-19 period, each percentage increase in SVI was found to correspond to a 161% increase in EMS rate (IRR 2.61, 95% CI [1.90, 3.59], *p* < 0.05) assuming all other factors remain constant. During the COVID-19 pandemic, each percentage increase in overall SVI was associated with a 192% higher EMS rate for probable asthma (IRR 2.92, 95% CI [2.12, 4.04], *p* < 0.05). The association between SVI themes and probable asthma EMS rate during the COVID-19 period showed some similarities with the pre-COVID-19 period. Specifically, socioeconomic status and housing type/transportation maintained comparable effects on EMS rate during both periods. However, there were notable changes during the COVID-19 period, with household characteristics becoming significantly associated with EMS rate and minority status more than doubling in effect. Controlling for rurality did not change any of our results (see Table 4).

**Table 4:** Negative binomial regression analysis for SVI and asthma-related EMS rates of children.

SVI^a^	Pre-COVID-19			During-COVID-19		
	IRR^b^	SE^c^	95% CI^d^ (Lower, Upper)	IRR	SE	95% CI (Lower, Upper)
Overall	2.61***	0.16	(1.90, 3.59)	2.92***	0.17	(2.12, 4.04)
SES^e^	2.60***	0.17	(1.87, 3.63)	2.39***	0.17	(1.70, 3.36)
Household characteristics	1.26	0.18	(0.87, 1.81)	1.77**	0.19	(1.22, 2.56)
Minority status	1.57*	0.23	(1.01, 2.48)	3.20***	0.23	(2.05, 5.01)
Housing type and transportation	2.61***	0.16	(1.90, 3.59)	2.92***	0.17	(2.12, 4.04)

a SVI: Social Vulnerability Index.

b IRR: Incident Rate Ratio.

c SE: Standard Error.

d CI: Confidence Interval.

e SES: Socioeconomic Status.

*Statistically significant (*p*-value <0.05). **Statistically significant (*p*-value <0.01). ***Statistically significant (*p*-value <0.001).

## Discussion

In county-level analyses set in Illinois, we found that overall SVI and most of its four themes are associated with the rate of EMS visits for probable asthma in both children and adults. In general, associations became stronger during the pandemic than before, especially among children. The current ecological study is the first to focus on social vulnerability at the county level as associated with an under-utilized data source- EMS visit rates. We found significant associations with SVI overall and also by theme, highlighting a distinct perspective on healthcare-seeking behavior for asthma-related emergencies before and during a public health crisis.

Asthma affects millions of individuals worldwide, wielding a notable impact on disadvantaged communities (45, 46). Sudden asthma attacks and subsequent emergency department visits occur disproportionately in vulnerable groups, particularly individuals with limited income, members of racial or ethnic minorities, and those with restricted access to healthcare services (10, 47). Research has confirmed the disparities in asthma management and healthcare utilization among these demographics (48). Prior studies have demonstrated a higher number of asthma-related ED visits in underprivileged areas (35, 49, 50). Our results were consistent with previous studies of social vulnerability and both emergency department visits and hospitalizations (51). In particular, Nayak et al. found associations between the SVI and emergency department visit rates for asthma (35). Yet, no previous study has examined the association between SVI and asthma-related EMS visits. As the SVI increases, so does the rate of EMS visits for probable asthma. This points to more asthma-related emergencies in counties with greater social vulnerability, which relates to both higher prevalence and poorer control of asthma.

In Illinois, the prevalence of asthma was consistently higher (8.2–8.7%) than the national average (7.7–7.8%) between 2018 and 2021, especially in Cook County, which contains the city of Chicago (1, 52–54). Illinois counties with higher overall SVI rates displayed a moderate but highly significant correlation with increased asthma EMS rates in our study (*r* = 0.42, *p* < 0.01) (30). This suggests a noteworthy correlation between county vulnerability and higher rates of asthma-related EMS visits.

Prehospital management of acute exacerbation of asthma symptoms, namely asthma attack, is a crucial component of EMS (45). So far EMS data are underutilized in the literature. In a recent report by the Respiratory Health Association (RHA), the association between asthma-related EMS and SVI has been referenced but not examined in detail (55). In addition, by using EMS rates as the outcome measure, this study contributes critical insights into a real-time indicator of acute healthcare needs. This was particularly relevant during the COVID-19 pandemic, where hospital overcrowding and fear of COVID-19 exposure likely shifted patient reliance toward pre-hospital care systems, as people tried to avoid visiting providers (36, 37).

Using a county-level indicator of social vulnerability may add community context, apart from, or in addition to individual characteristics. No ecological study of associations between asthma related EMS rates and SVI has been publixhed, and there are only a few studies of EMS rates and individual characteristics. A cross-sectional study conducted in Houston, Texas of EMS ambulance-treated cases of asthma found that factors such as earning less than $10,000 per year, being black, being female, and having less than a high school diploma were associated with living in areas with high rates of EMS ambulance treatment for asthma (56). A state-wide study in Florida, limited to cases of pediatric asthma with an average age of 9 years, found that 49% of the cases were African American and that high numbers were seen in four rural counties with a high proportion of African Americans (57). However, these studies have only examined a few of the 16 characteristics captured by the SVI.

Our findings among both children and adults, both before and during the COVID-19 pandemic confirm previous literature on socioeconomic status and asthma. The socioeconomic status theme within the SVI includes components, such as the proportion of the population below 150% of the poverty level, the proportion unemployed, without health insurance, without a high school education, and with a high housing cost burden, relative to their resources. The relationship between asthma-related emergency department visits with socioeconomic status has been documented. According to the American Community Survey, approximately 11.9% of the population in Illinois was living in poverty in 2020 (58). Numerous national and international studies have supported the idea that individuals from lower socioeconomic backgrounds are more likely to require emergency management, consistent with our study’s findings (47). Similarly, a recent cohort study with more than 30,000 workers and others present at the site of the World Trade Center disaster highlighted the link between socioeconomic status, more specifically, lack of money or health insurance, and asthma-related ED visits. Based on these findings, Alper et al. emphasized the necessity of identifying vulnerable populations who are more likely to have poorly controlled asthma, to alleviate the burden on hospitals during or after a catastrophe (59).

In the SVI the racial and ethnic minority status theme is indicated by the proportion of the population which is non-white or Hispanic. The association between race and asthma has been long established (60, 61). Higher prevalence, emergency visits, and mortality in Blacks, Hispanics, and other minorities due to asthma have been observed (48, 62, 63). Our analyses of EMS association with this theme were not statistically significant for adults before or during the COVID-19 pandemic but were for children, among whom the association increased during the pandemic. Further research is needed to ascertain why the association increased so dramatically in children during the pandemic. A possible explanation for this finding may be the reduced rate of nationwide asthma attacks among non-Hispanic white children (from 6.8 to 5.5%), along with a 6% higher rate of overall asthma prevalence in Illinois during the COVID-19 pandemic (54, 64, 65). Another possibility is that upper respiratory tract viruses serve as a common trigger of asthma, affecting black and Latinx children more than non-Hispanic whites (66–68). Hence, during the COVID-19 pandemic, the early infection phase of the SARS-CoV-2 affecting the upper respiratory tract could serve as the potential trigger for asthma (69).

Another SVI theme is household characteristics, which consist of the proportion of people aged 65 or older, children aged 17 or younger, disabled civilians, single-parent households, and those with English language proficiency. Having a child, an older adult family member, or someone with a disability in the family may increase the likelihood of a sudden emergency condition. As of 2020, about 38.8% of the population of Illinois are either 17 years old or younger, or over 65 years old. Additionally, 7.5% of the population below 65 years old have one or more disabilities (58). Older adults and young children are more likely to visit the emergency room due to asthma compared to other age groups (48, 70). A recent study in the US with more than seventy thousand children discovered that children with developmental disabilities have a higher prevalence of asthma, more than twice that of children without any disability (71). Our study found that among adults, these factors contributed to a 72% higher rate of EMS visits before COVID-19 (IRR 1.72, *p* < 0.05). During the COVID-19 pandemic, it reduced to a 66% higher rate (IRR 1.66, *p* < 0.05). However, among children, during the pandemic, the increase was 77% (IRR 1.77, *p* < 0.05).

The fourth theme contained within the SVI is housing type and transportation, which includes the proportion of the population living in multi-unit homes, mobile homes, or group quarters, the proportion living in crowded conditions, and the proportion with no vehicle. Some of these factors are known to be associated with asthma, particularly living in public housing or subsidized apartments, in which environmental accommodations depend on the cooperation of the landlord (72–74). A recent scoping review of 33 studies compiled evidence of significant associations between features of a house, such as location, accommodation, inhabitation, and asthma (75). Pollutants, both indoor and outdoor, play a significant role in triggering an asthma attack, as do unrestricted green areas through pollen (76–78). While the number of people in a family does not affect asthma, the number of people in a city does (79). Crowded cities have more EMS visits, especially from disadvantaged populations (80, 81). Illinois, having one of the most populous counties and densest cities in the country, has had issues with air pollution (82). Living in a place with more dampness and less air circulation, like a mobile home or a basement, can worsen asthma symptoms (83, 84). Our findings for the housing characteristics and transportation theme are consistent with all these results. The associations did not change much from pre-COVID-19 to during the pandemic but were stronger for children than for adults.

We adjusted for rurality in our negative binomial models on the basis of the idea that it may affect the frequency of EMS visits and because varying distances to hospitals in rural versus urban areas may affect access. There are even a few rural counties in Illinois with no hospital at all. However, the results of models containing this covariate were virtually the same, indicating that rurality, as defined by IDPH, is not a potential confounder of the association between SVI and EMS rates.

## Strengths and limitations

This study’s strengths include the examination of an underutilized outcome variable, asthma-related EMS rates in relation to a widely used indicator of community social vulnerability with 16 components, both before and during a public health crisis, among both children and adults. However, it does have some limitations.

Because this is an ecologic study conducted at the county level, we cannot conclude from this study alone that the associations are also operative at the individual level. However, several similar associations have been reported at the individual level in multiple cross-sectional and cohort studies. Nevertheless, it cannot be assumed that any associations shown in this study are causal.

Although the precision of the definition of a probable asthma case improved between version 2.2.1 and version 3.4 of EMS data, it is possible that some cases were misclassified. Both versions required at least one administration of inhaled albuterol, however, which minimizes this possibility.

Though SVI is regarded as an effective tool to identify disparities in emergency management quickly, its validity has sometimes been questioned (85). Since it has predetermined indicators, the static nature might not reflect changes or evolving dynamics within a community, making it less useful for rapidly changing situations (86). However, the period of time investigated in this study was relatively short, and analyses were stratified by period.

Factors affecting vulnerability can vary greatly based on cultural, geographical, and sociopolitical contexts, and census-dependent SVI at a micro-level might not accurately reflect the local environment (87). For example, living in a multi-unit structure may not reflect a vulnerability in a large city in the same way that it might in a small rural town. An exposure-specific, multi-dimensional, evolving, and adaptable index may serve better in identifying and following up on vulnerable populations requiring emergency asthma management. However, the SVI has become one of the most commonly used indices, which facilitates comparisons with previous and future studies.

## Conclusion

This study is the first to examine associations between social vulnerability and EMS rates for probable asthma on the county level and sheds light on the critical issue of asthma management and control within socially vulnerable communities, particularly across the COVID-19 pandemic. It reveals a significant correlation between high levels of social vulnerability and increased rates of asthma-related EMS visits in Illinois counties. Additionally, the study stresses the significance of housing and transport conditions and environmental factors in asthma exacerbations, accentuating the need for targeted and elaborate intervention strategies to reduce asthma burden. Considering a global emergency, the study translates into a reflection of the extended necessity of asthma emergency management before and during the crisis.

These findings offer valuable insights into the complex interaction of social, environmental, and healthcare factors in asthma management. Policymakers should consider identifying areas with varied vulnerability levels for not only asthma but also other chronic diseases. It will allow them to allocate resources better to improve housing conditions, enhance access to healthcare services, and address environmental triggers, particularly in high-SVI regions. To do so, EMS data can serve as a valuable indicator for identifying at-risk populations and directing preventive care efforts. Programs aimed at improving asthma management, such as community-based asthma education and outreach initiatives, are essential. Moreover, integrating SVI into public health decision-making can help accelerate resource allocation where it is most needed.

Although this study’s ecological design limits the ability to examine individual-level risk factors, in some studies, community-level indicators have increased the risk of negative outcomes beyond the influence of individual factors alone (88). Future research could include both individual-level data along with community-wide factors to investigate this possibility in relation to asthma.

Future studies may also explore the interplay between asthma prevalence, poor control, and EMS utilization rates, examining the type and location of care provided during EMS responses (either on-site or in the ambulance on the way to the hospital). Other studies may make use of both the SVI and additional measures of social vulnerability or area deprivation. Comparative analyses across different geographic locations may also offer a more comprehensive understanding of the multifaceted influences on asthma-related emergency outcomes. Longitudinal studies are needed to assess the impact of interventions targeting socially vulnerable populations on asthma outcomes and to refine predictive models for emergency asthma care. Persistent monitoring through adaptive indices will help identify temporal trends and adjust interventions to enhance the resilience of healthcare systems and increase health equity.

## Data Availability

Publicly available datasets were analyzed in this study. This data can be found here: CDC/ATSDR SVI Data and Documentation Download of Place and Health of ATSDR (Agency for Toxic Substances and Disease Registry) repository, https://www.atsdr.cdc.gov/placeandhealth/svi/data_documentation_download.html. The datasets of EMS are available from IDPH but restrictions apply to the availability of these data, which were used under license for the current study, and so are not publicly available. Data are however available from the authors upon reasonable request and with permission of IDPH.
